# Molecular consequences of mitochondrial replacement may be masked from organismal traits in *Tigriopus californicus*

**DOI:** 10.1371/journal.pone.0335181

**Published:** 2025-10-24

**Authors:** Jacob R. Denova, Ben A. Flanagan, Murad Jah, Scott L. Applebaum, Suzanne Edmands

**Affiliations:** 1 Department of Biological Sciences, University of Southern California, Los Angeles, California, United States of America; 2 Environmental Studies Program, University of Southern California, Los Angeles, California, United States of America; Centre National de la Recherche Scientifique & University of Nice Sophia-Antipolis, FRANCE

## Abstract

Mitochondrial replacement therapy (MRT) presents a promising preventative measure to combat mitochondrial diseases. However, the long-term consequences of disrupting mitonuclear coevolution at both the molecular and organismal levels remain understudied. Data on sex-specific effects are also lacking despite predictions that males may be especially vulnerable to mitochondrial replacement. To address this, we used backcrossed lines of the copepod *Tigriopus californicus* to produce offspring with nuclear genotype contributions from two populations and a mitochondrial genotype from a third, separate, population. When compared to hybrid controls with mitochondrial genotypes that matched the maternal nuclear genotype but not the paternal, these “three-parent offspring” did not significantly differ in lifespan or routine metabolic rate. While these organismal-level traits showed no effect, molecular metrics of mitochondrial health revealed consequences of mitochondrial replacement. Oxidative DNA damage, measured by 8-hydroxy-2’-deoxyguanosine content, was higher in three-parent offspring, and mitochondrial DNA content was lower than in hybrid controls. While differences between sexes were present in some traits, sex did not interact with mitochondrial replacement for any of these metrics. Although these results could be due either to donor mitochondrial DNA matching neither of the nuclear parents, or to deficits in the donor mitochondrial DNA itself, they highlight the importance of considering molecular level consequences of mitochondrial replacement that may be masked at the organismal level when evaluating the health impacts of this treatment.

## Introduction

Mitochondrial diseases, diseases caused by mutations in maternally inherited mitochondrial DNA (mtDNA), are a debilitating class of human conditions with current treatments focused on symptom management rather than addressing the underlying genetic defects [1,2]. Mitochondrial replacement therapy (MRT) offers a promising solution by replacing defective mtDNA with healthy donor mtDNA, effectively preventing transmission of these diseases [3,4]. The technique produces “three-parent offspring” with nuclear DNA (nDNA) from both parents and mtDNA from a donor, and the methods are well developed, producing viable offspring across a wide variety of mammal species including humans [5,6]. Despite this, mitochondrial replacement is not widely accepted as a solution for mitochondrial diseases [6].

One concern limiting the use of MRT is the potential for mitonuclear incompatibility. The mitochondrial and nuclear genomes must interact harmoniously to support essential processes such as cellular respiration and other integral mitochondrial functions [7,8]. Strong evidence across a variety of animal taxa, including humans, suggests these vital interactions drive the coevolution of the two genomes, ensuring their compatibility over time [9–14]. Despite the continual reshuffling of nuclear haplotypes through recombination and biparental inheritance, mitonuclear coevolution can emerge because selection consistently favors nuclear variants that function optimally with the maternally inherited mtDNA, allowing compatible alleles to accumulate over time. Breaking up this coevolved system, whether through naturally occurring hybridization [15] or artificial interventions like MRT [3,16], results in mitonuclear mismatch where potential complications can arise. In this terminology, “mismatch” is used as a practical descriptor of coancestry patterns, rather than a presumption of functional incompatibility. Under normal sexual reproduction, offspring have mtDNA that is partially matched to the nuclear genome because the mother provides both the mtDNA and approximately half the nDNA. An offspring produced by MRT has mtDNA that is completely mismatched to its nuclear genome because its mtDNA descends from neither nuclear parent. This means the nuclear and mitochondrial genomes no longer share recent coancestry and the offspring will not benefit from any mitonuclear coadaptation resulting from maternal inheritance. This risk is heightened by evidence that mitonuclear incompatibilities tend to be recessive [17–19], meaning that the mitochondrial haplotype has deleterious interactions with homozygous recessive nuclear genotypes. In normal sexual reproduction, compensatory mutations in coevolved mtDNA can allow such deleterious recessive alleles to persist. MRT increases the risk of unmasking these recessive incompatibilities by creating novel mitonuclear combinations that have not undergone selective purging. This is particularly problematic in later generations, beyond the initial MRT offspring, when nuclear variants are more likely to become homozygous against the donor mtDNA background.

Mitonuclear disruption caused by MRT may impact organismal traits such as longevity and metabolic rate, as well as related molecular traits such as oxidative damage and mtDNA content. The link between mitochondrial dysfunction and aging, highlighted by the free radical theory of aging [20], suggests that mitonuclear mismatch may increase reactive oxygen species (ROS) and exacerbate oxidative stress, leading to DNA damage and shortened lifespan [21–23]. For example, mismatched genomes have been shown to increase hydrogen peroxide production, accelerating oxidative damage with age [22]. MtDNA content is also a potential biomarker of health [24], although its associations vary: elevated levels in mismatched systems correlate with DNA damage [22], while low levels are linked to aging phenotypes [25]. Metabolic rate also shows varied effects, as mismatch can induce mitochondrial inefficiency that can either elevate or suppress metabolism [26]. Despite the interconnectedness of these traits, impacts at the molecular level are not always apparent at the organismal level. Compensatory mechanisms and alternative pathways can mask consequences of a treatment [27] underscoring the need to assess both organismal and molecular outcomes when evaluating the long-term risks of MRT.

Concerns of sex-specific effects of mitochondrial replacement also hinder the procedure from being widely accepted. The Mother’s Curse hypothesis predicts the accumulation of male-harming mtDNA mutations, because maternal inheritance of mitochondria makes these mutations invisible to selection if they are neutral or beneficial in females [28,29]. While these male-harming mtDNA mutations can be masked by compensatory nuclear mutations, they can be unmasked when mtDNA is placed on a new nuclear background by MRT, potentially leading to greater deleterious effects in males [30]. Support for the hypothesis has been mixed, with some studies even showing the unexpected result of more deleterious effects of mitonuclear mismatch in females [31,32]. Additionally, asymmetric inheritance of mitochondria and sex chromosomes in species with chromosomal sex determination may further complicate experimental estimates of sex-specific conflicts due to the co-segregation of sex chromosomes and the mitochondrial genome [33]. These results highlight the importance of investigating sex differences in mitochondrial function and their implications for MRT outcomes.

The copepod *Tigriopus californicus* is an ideal model system to study the effects of mitochondrial replacement. This species exhibits high population structure with mtDNA divergence exceeding 20% between reproductively compatible populations [34–36]. Evidence of mitonuclear coevolution in isolated populations [36] further supports its suitability for testing the consequences of mitonuclear disruption. While mitochondrial divergence in *T. californicus* is vastly greater than that estimated in humans (~ 0.13%) [37] there is much evidence that humans also have co-evolved nuclear and mitochondrial alleles [11,12,38], with human cybrid lines combining one nuclear genome with different mitotypes revealing incompatibilities [38]. Crosses between genetically distant *T. californicus* populations can therefore be used to understand mechanisms underlying mitochondrial replacement and to explore a “worst case scenario”. *T. californicus* also has the advantage of a short generation time, which can be further reduced by raising the temperature, enabling lifespan measurements within practical timeframes [31,39]. In addition, this species has polygenic sex determination [40] eliminating the confounding effects of sex chromosomes. Despite the lack of sex chromosomes, the species exhibits strong evidence for sex specific mitochondrial and mitonuclear effects [41,42]. These traits make *T. californicus* a powerful system for investigating the consequences of mitochondrial replacement.

**Fig 1:**
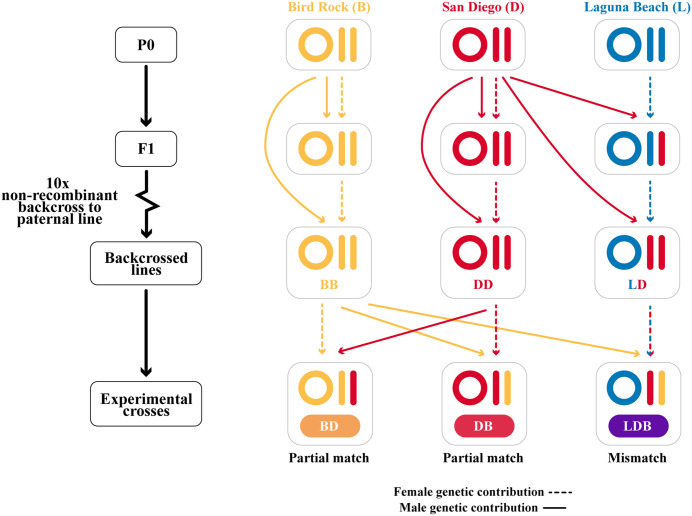
Inbred lines from three populations (Bird Rock: yellow; San Diego: red; Laguna Beach: blue) were crossed to produce hybrids that were backcrossed 10 times to the paternal line creating three non-recombinant backcrossed lines. These lines were then hybridized to produce three experimental crosses which share the same nuclear background but have different mtDNA: BD, DB, and LDB (first initial is the mitochondrial type). BD and DB are “two parent offspring” with partial mitonuclear match, while LDB are “three parent offspring” with full mitonuclear mismatch. Lifespan, routine metabolic rate, 8-OH-dG content, and mitochondrial content assays were conducted on the experimental crosses. Circles represent mitochondrial genomes, and bars represent parental nuclear genome contributions. Coloration of lines represents parental contributions.

In this study, we used *Tigriopus californicus* lines subjected to a rigorous breeding regime designed to mimic the mitonuclear mismatch present in MRT. Unlike studies focusing on standard hybrids [43], our approach more closely replicates mitochondrial replacement by incorporating nuclear genetics from two populations and mitochondria from a third population that is substantially divergent from both nuclear parents (Fig 1). These individuals represented our mismatched MRT individuals and were compared to partially matched individuals that inherited the maternal population’s mtDNA and half of their maternal population’s nDNA, mirroring natural reproduction. To uncover the long-term effects of MRT, we tracked individuals and measured lifespan alongside key traits related to ageing and mitochondrial health. Specifically, we assessed oxidative DNA damage (8-hydroxy-2’-deoxyguanosine, 8-OH-dG) to test how mitochondrial replacement affects ROS production and management in ageing individuals, mtDNA content to explore its relationship with health outcomes, and oxygen consumption by measuring routine metabolic rates to determine whether metabolism varies under mitochondrial replacement. If disrupting the coevolution between the mitochondrial and nuclear genomes through MRT is detrimental, we would expect to see adverse effects in our mismatched experimental animals compared to the “normal,” partially matched controls. Specifically, we predict both organismal level effects (reduced lifespan and altered respiration) and cellular level effects (increased oxidative damage and changes in mtDNA content). By using an organism with polygenic sex determination, we avoided confounding effects of sex chromosomes, enabling a clearer assessment of the direct sex-specific impacts of mitochondrial replacement.

## Methods

### Backcrossed lines

*Tigriopus californicus* were sampled from supralittoral pools at Laguna Beach, CA (L: 33.54 N, 117.79 W), Bird Rock, CA (B: 32.82 N, 117.27 W), and San Diego, CA (D: 32.7460 N, 117.2551 W)(Specimens were collected under California Department of Fish and Wildlife Scientific Collecting Permits (SC-007243, SC-240780006)). These population samples, as well as all derivative lines, were maintained in stable conditions in a 20°C incubator with a 12-hour day/night cycle. From each of the three locations a single isofemale line was initiated from a single female fertilized by a single male and these three isofemale lines (B, D and L; Fig 1) were maintained for a minimum of five years before backcrosses began. The non-recombinant backcrossed lines used in this study (Fig 1) were created by Watson et al. 2022 [31] through an 18-month breeding regime in which isofemale lines were hybridized to create F1 hybrids, and female hybrids were backcrossed to the paternal line for 10 generations. Because female meiosis is non-recombinant in Tigriopus [44], maternal nuclear alleles are transmitted intact in these backcrosses, ensuring that non-coancestral nuclear backgrounds are faithfully maintained in mitonuclear hybrids. Whole-genome sequencing of pooled samples from each of the backcrossed lines verified their expected mitonuclear genotypes [31 and related unpub data]. Potential paternal mtDNA leakage was found to be very low (0.02–0.28%), a level that cannot be distinguished from sequencing error (0.1–1%). From the few remaining extant lines from this earlier study, three were chosen for this experiment: BB and DD are fully matched controls, while LD results is a mismatched line with L mtDNA and D nDNA. Whole-genome sequencing of pooled samples from each of the three backcrossed lines verified their expected mitonuclear genotypes. [31]These three backcrossed lines were maintained for more than 20 generations after the original study. The 12S mitochondrial gene (forward primer 5’-CCAAGATACTTTAGGGATAACAGC-3’, reverse primer 5’-CTGTTCTATCAAAACAGCCCT-3’) was then sequenced in the three lines, confirming that there had been no mixing between lines.

### Experimental crosses

These backcrossed lines were crossed to create three sets of offspring (Fig 1). Each resulting cross has the same nuclear background, but a different mitochondrial genome. Reciprocal crosses between DD and BB lines generated two sets of two-parent offspring (BD and DB, with the mitotype population listed first), while LD females were crossed with BB males to create three-parent offspring (LDB) simulating mitochondrial replacement. Note that the partially matched two-parent offspring (BD and DB) have mtDNA that is identical to the maternal line and highly divergent from the paternal line, while the three-parent offspring (LDB) have “donor” mtDNA (L) that is mismatched for both nuclear parents – it is highly divergent from the maternal nuclear line (D) and mildly divergent from the paternal nuclear line (B) (Table 1) [31].

**Table 1:** Mitochondrial DNA divergence between the inbred lines (D, B and L) used in this study.

	D-B	L-D	L-B
MtDNA divergence	9.6%	9.5%	1.9%

Unmated females were selected by separating clasped pairs in which adult males clasp virgin females [45]. All experimental crosses and lifespan measurements were maintained in 25°C incubators with 12-hour day/night cycles. Crosses were established by placing one male and one female from the desired populations in each petri dish with 4 mL of culture medium consisting of 0.1 g of ground Spirulina (Nutraceutical Science Institute, USA) and 0.1 g of ground Tetramin fish food (Tetra Holding Inc., USA) per liter of 3x- filtered seawater from the USC Wrigley Marine Science Center (Catalina Island, CA, USA). Petri dishes were checked daily, and males were removed once the female in the dish was observed with an egg sac. Females were removed once larvae were observed, which was designated day 0 for the offspring.

Offspring were raised individually in isolated wells once they entered the juvenile stage (~ 14 days old) to prevent mating between siblings. Sex was determined beginning at 16 days of age, as sex in this species cannot be reliably determined based on morphology until adulthood, when males develop characteristic geniculate first antennae. In families with large clutches, offspring were selected haphazardly for this study. Individuals were isolated in separate wells of 24-well plates containing 3x- filtered seawater with the same starting ration of food as mating plates, which was rehydrated as needed with DI water to compensate for evaporation. Food was replenished once weekly using ~0.02 µg each of ground Spirulina and ground Tetramin per individual.

### Lifespan data

Beginning at 16 days of age, individuals were monitored three times a week (Monday, Wednesday, and Friday) until death. At 49 days of age individuals of each sex were removed from each cross for use in other assays and censored from the lifespan data to avoid bias in the survival analysis. Lifespan was measured in a total of 1,212 animals.

### Relative mtDNA content

One subset of animals taken at 49 days of age was used for estimates of relative mtDNA content. These animals were individually frozen and held at −80°C until assayed. DNA was extracted from individuals by incubation at 65°C for 1 hour in 50 ml proteinase K (200 mg ml^-1^) cell lysis buffer (10mM TRIS, 50mM KCl, 0.5% Tween 20, at pH 8.8), followed by 15 minutes of denaturation at 100°C before refreezing at −80°C. mtDNA content was then estimated through two sets of qPCR per individual targeting the single-copy nuclear gene *AtpC* and the single-copy mitochondrial gene *Atp6*. This approach normalized mtDNA content to nDNA content, to account for individual size variation. Targets were amplified using primers designed by Flanagan et al. [41] (*AtpC*-F 5’-CCAAGTTCATCGGAGCTGGT-3’; *AtpC*-R 5’-TACGGGCGTAACCGATGATG-3’; *Atp6*-F 5’-TGAGAACCAGAATGAACGGCT-3’; *Atp6*-R 5’-AGGGTCTTCTCGTCCCTGAA-3’).

Protocols for qPCR were optimized using a five-fold serial dilution of template DNA to ensure primer efficiencies between 90% and 110% with an r^2^ > 0.95. Reactions were run using an Agilent AriaMx Real-Time PCR System (Agilent) with a reaction mixture consisting of 10 µL Brilliant III Ultra-Fast SYBR® Green qPCR Master Mix (Agilent), 2 µl 0.002X reference dye (Agilent), 5 µl 0.5 μM primers and 2 µl DNA lysate. The protocol included initial denaturation at 95°C for 3 minutes, followed by cycles of denaturation at 95°C for 15 seconds, annealing for 15 seconds at 65°C for the nuclear target and 55°C for the mitochondrial target, followed by 60°C extension for 15 seconds for the nuclear target and 30 seconds for the mitochondrial target. The cycles were repeated 40 times followed by a melt period to ensure specificity. Ct values were obtained for each individual using regression analysis included in the AriaMx software. All samples were assayed in triplicate. Samples with Ct values differing by more than one cycle between replicates were rerun. Using the average of each of both mtDNA and nDNA Ct values, mtDNA content as a ratio of mtDNA copies to nDNA copies was calculated using methods previously described in Rooney et al. 2015 [46].

### Oxidative DNA damage

Another subset of animals frozen at −80°C at 49 days of age was used to assay oxidative DNA damage by measuring 8-hydroxy-2′-deoxyguanosine (8-OH-dG) content as an estimate of guanosine oxidation. An enzyme-linked immunosorbent assay, ELISA, was used to measure this oxidative damage (Cayman Chemical cat. 589320). Methods identical to the “Relative mtDNA content” section were used to extract DNA from individuals using a proteinase K lysis buffer extraction. However, due to the sensitivity of the assay, individuals further underwent a phenol-chloroform extraction to maximize DNA yields. First, 100 µl of phenol-chloroform (pH 8.0: VWR) was added to the product of each sample’s lysis extraction. This mixture was briefly vortexed and then centrifuged for 5 minutes to separate out an aqueous layer. This layer was decanted off and preserved before 80 µl of 0.5x TE (pH 8.0) was added to the initial phenol-chloroform mixture. The process of vortexing, 5 minutes of centrifugation, and decanting off the aqueous layer was repeated with the decanted solution being added to the initial collection of the aqueous layer. This product was then taken and 500 µl of ice-cold 95% ethanol was added, followed by 1 µl of GlycoBlue (Invitrogen) and 78 µl of 3 M NaOAc. The samples were then inverted and incubated at −20°C for 1 hour. After this, the samples were centrifuged for 30 minutes to form a solid pellet, and the remaining solution was discarded. The pellet was rehydrated using 300 µl of ice-cold 70% ethanol and centrifuged again for 10 minutes to reform the pellet. The solution was once again decanted off and the samples were then dried completely using a vacuum centrifuge. Each sample had 60 µl of molecular grade water added to resuspend the DNA which was quantified using a Qubit™ 3 Fluorometer (Invitrogen) with a Qubit™ dsDNA HS Assay Kit (Invitrogen). Due to the amount of DNA needed for the ELISA protocols, three individuals of each like sex and cross were pooled to ensure readings fell within the standard curve produced by the assay. After they were pooled, samples were treated with P1 nuclease (New England Biolabs) then rSAP (New England Biolabs). Pooled samples were then used in the 8-OH-dG damage ELISA that was performed following manufacturer’s protocols. Each pooled sample had four technical replicates run with two being 1x and two being 0.5x concentration of sample. These replicates were averaged and results normalized to the total DNA of each sample.

### Routine metabolic rate

A final subset of individuals was taken between ages 47 and 49 days. Males and females from all three crosses were taken from their ambient conditions and briefly placed on filter paper. Despite being unmated, females produced unfertilized egg sacs and these were gently removed. Each individual was then placed in a well of an optical microplate respirometer (24-well Microplate Core system, 80 ml wells, Loligo Systems, Viborg, Denmark) containing 0.2 µm filtered, autoclaved, air-saturated seawater. Of the 24 wells, two did not have a copepod added and served as controls for any background oxygen consumption. Wells were sealed, kept in darkness at a steady 23°C, and allowed 25 minutes to reach equilibrium before initial oxygen concentration was recorded. Oxygen concentration was then recorded every 5 minutes for 185 minutes, with the slope of the resulting line representing an individual’s oxygen consumption rate. This measurement gave an estimate for routine metabolic rate, as animals’ behavior was intentionally not modified by any experimental factors. For the duration of the assay, conditions remained normoxic and no mortality was observed in individuals tested.

Immediately following the respiration assay, individuals were frozen and stored at −80°C for protein content assays to correct for size. Total protein content was estimated using a bicinchoninic acid (BCA) microassay (Pierce Micro BCA Protein Assay, Thermo Fisher Scientific). Individuals were thawed and 350 µl of ultrapure water was added before they were homogenized on ice by sonication (VirSonic 100 Model #346411, Virtis) for 10 seconds at 7 watts RMS (Root mean squared). Two replicate samples of 150 µl were assayed according to manufacturer’s instructions with a total assay volume of 300 µl. Samples were then incubated for 60 minutes at 60°C and a standard curve created using bovine serum albumin provided by the manufacturer. This curve was used to calculate total protein content (µg ind^-1^) of individual copepods from sample absorbance values measured at 562 nm in a SpectraMax plate reading spectrophotometer (Molecular Devices, LLC, San Jose, CA). Respiration rates were normalized to protein content to account for size differences among individuals.

### Analysis

All data were visualized and analyzed in R version 4.4.0 [47]. Survivorship data were analyzed using Kaplan-Meier curves [48] to evaluate effects of cross and sex, and log-rank analyses to evaluate interactive effects of mitochondrial genotype and sex using the *survival* package [49]. Data were further analyzed using a Cox proportional hazards model to test for interactive effects of sex and mitonuclear mismatch on survival times. Data from qPCR, 8-OH-dG DNA damage, routine metabolic rate, and protein content assays were analyzed using generalized linear models fitted to lognormal and Gamma distributions with inverse link functions due to non-normality of the data. The best-fit distribution was selected using Akaike Information Criterion (AIC). Effects of cross, sex, and their interaction were tested under the selected model, with post-hoc pairwise comparisons, including correction for multiple tests, performed using Tukey-corrected least-square means using the *emmeans* package in R [50].

## Results

### Survivorship

**Fig 2:**
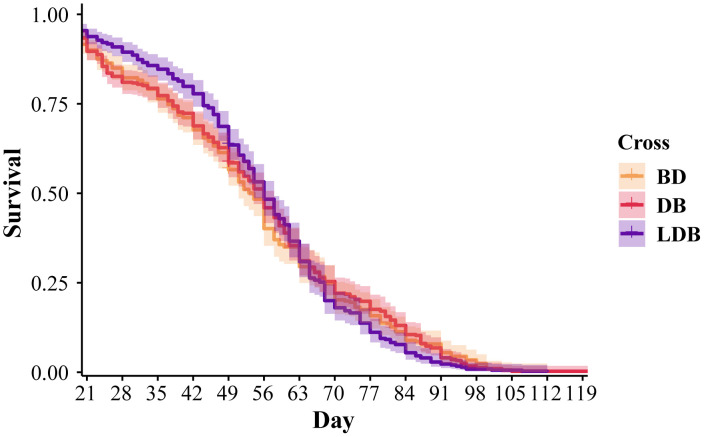
Survival proportions (±SE) of the three crosses of *T. californicus* starting at 16 days old after animals reached sexual maturity. No significant differences were detected between the crosses using pairwise log-rank analyses or Cox proportional hazards modeling.

The lifespan of 1,212 individuals across the three treatments was measured (BD = 356; DB = 458; LD = 398) with an additional 252 (84 from each line) censored for use in various assays. Among uncensored individuals, 922 were male and 290 were female (1,048 males and 416 females when including censored individuals). Log-rank analyses accounting for censoring revealed no significant differences in survival based on cross (χ^2^ = 0.2, df = 2, *p* = 0.9) (Fig 2) or sex (χ^2^ = 0.7, df = 1, *p* = 0.4). A Cox proportional hazards model was fit to the data to investigate potential interactive effect of cross and sex on survival (Table 2). The model showed no effects of cross (χ^2^ = 0.270, df = 2, *p* = 0.874), sex (χ^2^ = 0.801, df = 1, *p* = 0.371), or their interaction (χ^2^ = 1.37, df = 2, *p* = 0.505).

**Table 2:** Interactive effects of sex and cross on survival (from a Cox proportional hazards model) and on mtDNA content, oxidative DNA damage, and routine metabolic rate (from generalized linear models).

	d.f.	chi-sq.	*p*-value
*Survival*			
Cross	2	0.270	0.874
Sex	1	0.801	0.371
Cross: Sex	2	1.37	0.505
*mtDNA Content*			
Cross	2	12.0	0.002*
Sex	1	42.6	<0.0001*
Cross: Sex	2	0.604	0.739
*Oxidative DNA Damage*			
Cross	2	8.90	0.012*
Sex	1	0.237	0.626
Cross: Sex	2	0.944	0.624
*Routine Metabolic Rate*			
Cross	2	4.18	0.124
Sex	1	0.001	0.975
Cross: Sex	2	0.188	0.910

### Relative mtDNA content

**Fig 3:**
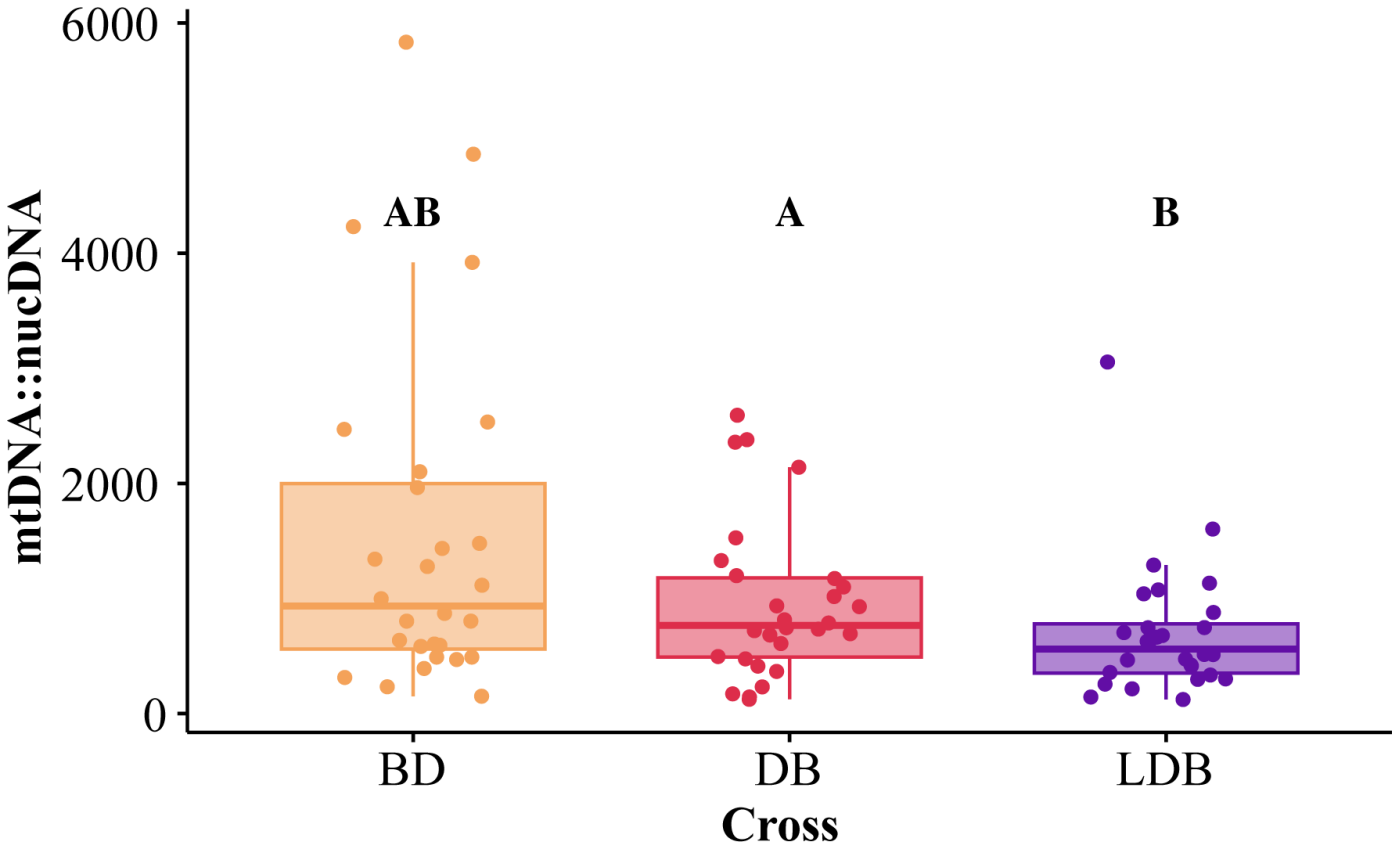
Mitochondrial copy number relative to nuclear copy number for each cross of *T. californicus.* Letters denote significance between log-transformed data from crosses with *post hoc* Tukey corrected *p*-values < 0.05.

From each cross, 14 males and 14 females were assayed for mitochondrial copy number relative to nuclear copy number. Relative mtDNA content measurements were log-transformed to improve model fit. Model results (Table 2) showed significant effects for both sex (χ^2^ = 42.6, df = 1, *p* < 0.001) (S1a Fig) and cross (χ^2^ = 12.0, df = 2, *p* = 0.002) (Fig 3), but not for their interaction (χ^2^ = 0.604, df = 2, *p* = 0.739). Females were shown to have higher mtDNA content (mean 1567 ± SE of 196 mitochondrial copies relative to nuclear copies) compared to males (mean 566 copies ± 54). Tukey-corrected pairwise comparisons revealed that the LDB cross had significantly lower relative mtDNA content than BD (BD mean 1536 copies ± 282, LDB mean 703 copies ± 110, p = 0.003), but no other pairwise comparisons were significant (DB mean 961 copies ± 130, BD/ DB *p* = 0.158, DB/ LDB *p* = 0.250).

### Oxidative DNA damage

**Fig 4:**
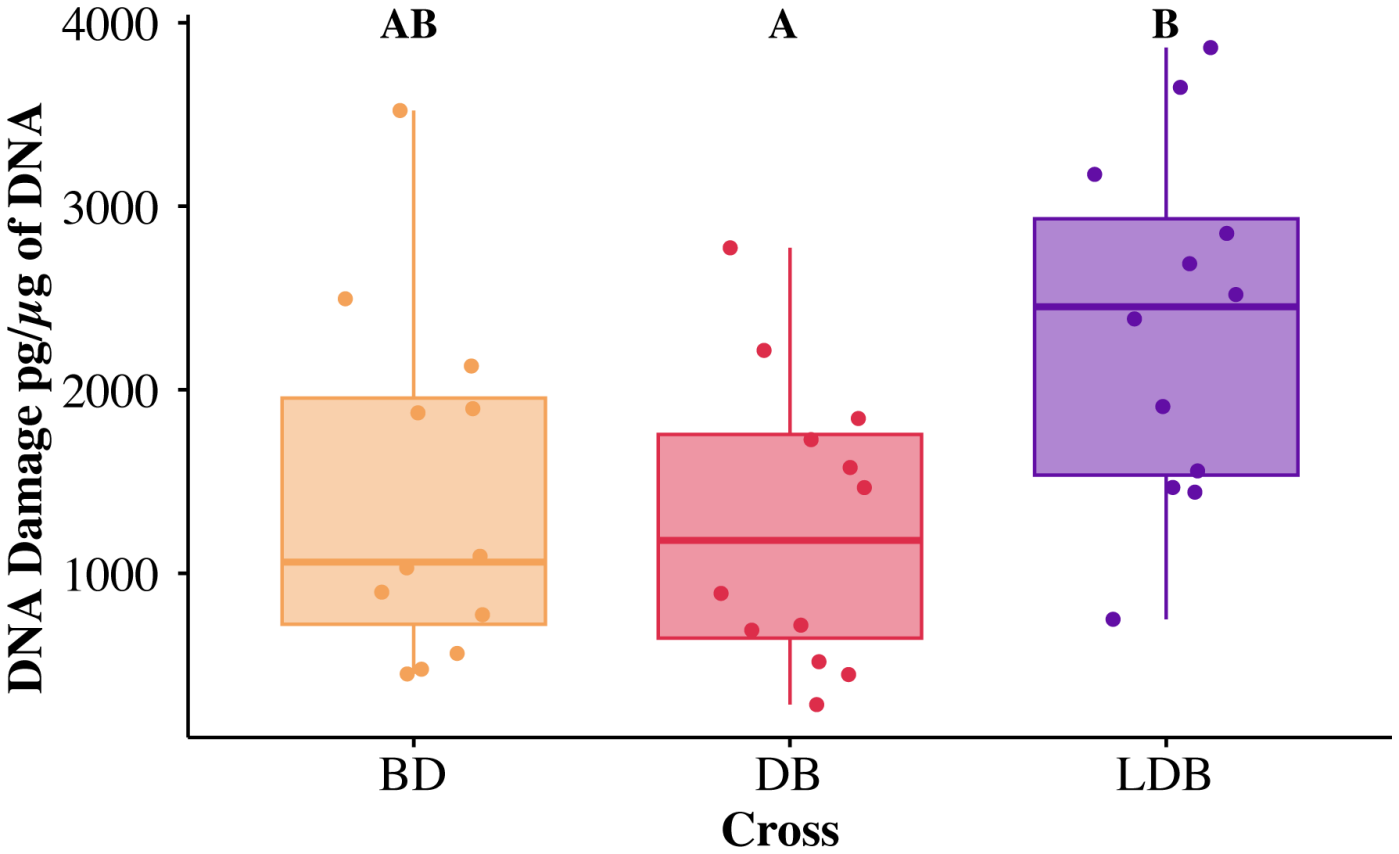
8-OH-dG DNA damage estimated by ELISA for each cross of *T. californicus.* Letters denote significance between log-transformed data from crosses with *post hoc* Tukey corrected *p*-values < 0.05.

Oxidative DNA damage was assessed by quantifying 8-OH-dG levels in six pooled samples per sex per cross, with each sample comprising three individuals. DNA damage was measured as picograms 8-OH-dG per microgram of DNA (pg/µg DNA) and log-transformed for optimal model fit. Model results (Table 2) showed no effect of sex (χ^2^ = 0.237, df = 1, *p* = 0.626) (S1b Fig), but a significant effect of cross (χ^2^ = 8.90, df = 2, *p* = 0.012) (Fig 4). No significant interaction was detected between sex and cross (χ^2^ = 0.944, df = 2, *p* = 0.624). Pairwise analysis with Tukey corrected *p*-values showed that the reciprocal controls were not significantly different from one another (DB mean 1262 ± SE of 227 pg/DNA (µg), BD mean 1433 ± 276 pg/DNA (µg), *p* = .876). However, LDB had significantly higher oxidative DNA damage than DB (LDB mean 2354 ± 276 pg/DNA (µg), *p* = 0.024) and trended towards higher damage than BD (*p* = 0.071).

### Routine metabolic rate

**Fig 5:**
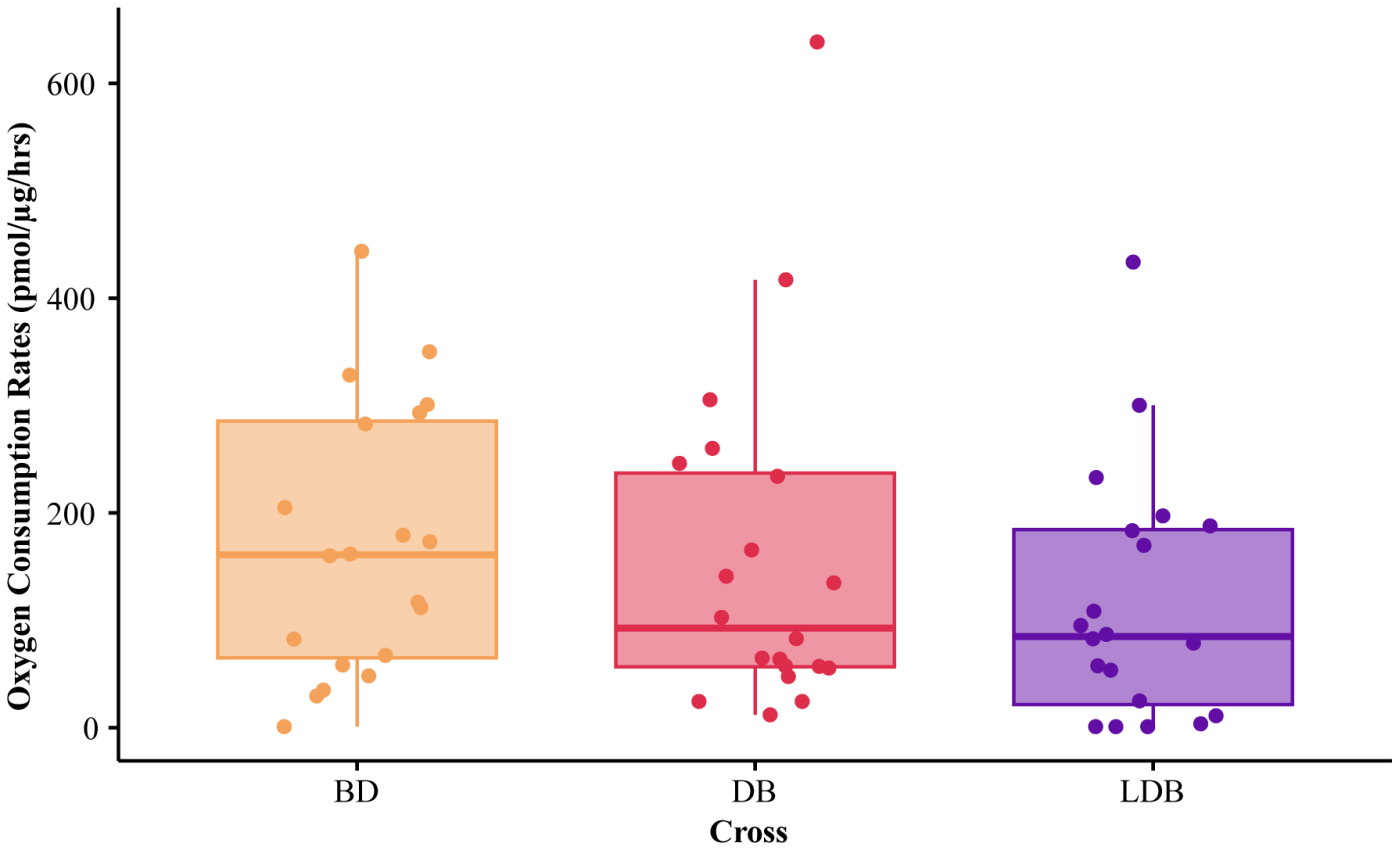
Routine metabolic rates estimated as oxygen consumed per picomole per hour then size corrected to protein content in *T. californicus.* No statistically significant differences from log-transformed data were uncovered between the three crosses.

Protein corrected routine metabolic rate (pmol O_2_/µg protein/hour) was assayed in 10 individuals of each sex from each cross. Model results (Table 2) for log-transformed data revealed no significant effect of cross (χ^2^ = 4.18, df = 2, *p* = 0.124) (Fig 5) or sex (χ^2^ = 0.001, df = 1, *p* = 0.975) (S1c Fig) nor was there evidence for an interaction between the two factors (χ^2^ = 0.188, df = 2, *p* = 0.910). Log-transformed protein values showed a significant effect of sex, with females having higher content (mean content 2.07 pg ± 0.0646 in males and 2.56 pg ± 0.0850 in females; χ^2^ = 20.2, df = 1, *p* < 0.001) (S1d Fig).

## Discussion

This study provides a novel perspective on mitochondrial replacement in an experimental invertebrate model. Our approach differs from human MRT in that mitonuclear divergence is substantially higher, and it also differs from typical F1 hybrid studies [43] in that the donor mtDNA matches neither parent, thereby creating a complete mitonuclear mismatch. To provide a broader understanding of the effects of mitochondrial replacement, we examined both organismal traits—lifespan and size-corrected routine metabolic rate—and molecular metrics directly related to mitochondrial function. Overall, significant phenotypic effects of different mitotypes on the same nuclear genetic background were found only at the sub-organismal level. Additionally, despite much evidence for sex-specific mitochondrial effects in this species [41,42,51] we found no evidence for any interactions between mitochondrial replacement and sex for any of the measured traits.

Effects of mitochondrial replacement are much debated, with some studies showing limited consequences [43] while others highlight significant risks [52]. Even with the high level of mitochondrial divergence in our crosses, we did not detect harmful effects of mitochondrial replacement on the organismal traits of lifespan or routine metabolic rate. The lack of lifespan effects contrasts with previous work in the system showing that interfering with mitonuclear coadaptation leads to impaired organismal traits, such as survival and development [53,54]. Mitonuclear incompatibilities have been shown to induce lower rates of oxygen consumption in *Drosophila* [22], but work to date on routine metabolic rate in *T. californicus* [51,55] has not shown significant mitonuclear effects. A contributing factor for the lack of organismal-level effects in our study is that the control lines were already hybrids with partially mismatched nuclear and mitochondrial genomes. If hybridization had already impaired function in these lines, lifespan may already be hindered and respiration rates may be near the lower limit of what is physiologically viable, regardless of whether mismatch is partial (as in controls) or complete (as in three-parent offspring). This baseline impairment could mask additional effects of mitochondrial replacement on routine metabolic rate.

Despite the absence of effects at the organismal level, mitochondrial replacement had significant consequences at the molecular level. Three-parent offspring had higher levels of oxidative DNA damage (8-OH-dG) than both controls (significant for one, Fig 4) and lower levels of mtDNA content than both controls (significant for one, Fig 3). While DNA damage was not linked to a shorter lifespan in our study, previous research showed that such damage accumulates with age in *T. californicus* [41] suggesting the three-parent offspring exhibited characteristics of accelerated molecular aging. Importantly, our measurement of 8-OH-dG content quantifies damage resulting from ROS, rather than ROS production itself. Whether this increased damage was due to increased ROS production or impaired ROS scavenging remains undetermined. Mitonuclear mismatch has been shown to upregulate genes related to ROS scavenging in natural bat populations [56], potentially as a compensatory mechanism for increased ROS production. However, our findings suggest that any compensatory ROS scavenging mechanisms in three-parent offspring were insufficient to prevent oxidative DNA damage when mitonuclear mismatch impaired electron transport chain function, leading to electron leakage during respiration.

The increased damage in the three-parent individuals aligns with a study of *Drosophila* showing elevated hydrogen peroxide production in lines harboring incompatible mitochondria [22]. Interestingly, our mtDNA content results diverged from this previous study: we found lower mtDNA content in our three-parent cross while Pichaud et al. 2019 found higher mtDNA content in mismatched *Drosophila*. This reduction may be caused by disrupted interactions between mtDNA and nuclear encoded factors underlying mitochondrial replication [57]. Our hybrid controls did not show a decrease in mtDNA content suggesting that the mitotype that is mismatched from both parents present in mitochondrial replacement cannot recover this efficiency to the same level achieved by partially matched individual controls. Reduced mtDNA content is concerning because this has been often associated with senescence [58,59]. While our three-parent offspring showed no evidence for decreased lifespan, this decrease in mtDNA content may impact aspects of fitness beyond lifespan.

Together, our findings across biological scales underscore the importance of investigating disruption of mitonuclear coevolution at multiple levels to gain a more comprehensive view of its effects. These results align with previous findings indicating that molecular level consequences of mitonuclear mismatch are not always reflected at the organismal level [27]. Similar discrepancies were documented in previous work on *T. californicus*, where evidence for the mitochondrially based “Mother’s Curse” hypothesis is robust for molecular traits such as gene expression but weaker for whole-organism phenotypes such as longevity and fertility [31,42]. The molecular-organismal disconnect in our study may result from compensatory mechanisms. Three-parent offspring exhibited lower mtDNA content but maintained similar oxygen consumption rates, suggesting possible upregulation of oxidative phosphorylation pathways or alternative metabolic pathways to compensate for mitochondrial deficiencies [27,60]. While these compensatory mechanisms may mitigate immediate fitness consequences, the lack of detectable organismal-level consequences does not mean that mitochondrial replacement carries no risks. Importantly, our study was conducted under constant, benign conditions, and organismal effects may manifest under stressful conditions imposing higher metabolic demands [61]. Further research examining how mitochondrial replacement affects organisms in high stress environments would provide valuable insights into potential consequences for human applications. Finally, mitochondrial replacement may also affect fitness-related traits outside of the traits we measured, as disruption of mitonuclear evolution has been linked to diverse phenotypic consequences in other studies [62–64]. Investigations into other organismal-level traits may reveal consequences of the molecular level effects we found in this study.

It should be acknowledged that due to the limited number of surviving lines from Watson et al. 2022 we were unable to create control lines testing for interactions between L mtDNA and only one nuclear background or the effect of L mtDNA alone. We therefore cannot distinguish whether differences in three-parent offspring were due to the mismatch between the L mtDNA and both nuclear parents or to the effects of the L mtDNA itself. Despite these confounding variables, our results have important implications for MRT, showing that donor mtDNA from an apparently healthy lineage induced subclinical markers of dysfunction that did not manifest as immediate organismal deficits.

Our work showed no evidence that mitochondrial replacement differentially affects males and females. While we observed sex-specific effects in certain measurements, these differences were consistent across treatments. Specifically, both protein content and mitochondrial content were female-biased, consistent with previous work in the system [41]. The detrimental effects of lower mitochondrial content in males may be masked under benign conditions, as shown in prior studies where males lived at least as long as females under benign conditions [41,65] but had shorter lives under nutritional stress [66]. It is also important to consider that the lack of differential sex effects in our study might be related to the fact that we are comparing three-parent hybrids to two-parent hybrids that already have mitonuclear incompatibilities. The pre-existing mitonuclear conflict in the two-parent controls may have masked any additional sex-specific vulnerabilities to mitochondrial replacement in the three-parent offspring. Regardless, our results challenge theoretical predictions that mitochondrial replacement should cause greater consequences in males [30]. This finding is particularly robust because our study system lacks sex chromosomes, eliminating a confounding factor that complicates similar research in organisms with chromosomal sex determination.

In conclusion, despite the absence of organismal-level effects, mitochondrial replacement showed consequences on *T. californicus* at the molecular level. Elevated oxidative DNA damage and reduced relative mitochondrial content suggest that disrupting mitonuclear coevolution in three-parent offspring induced underlying health consequences, even though these did not manifest as altered lifespan or routine metabolic rate. Additionally, we found no evidence that mitochondrial replacement disproportionately affects males compared to females in a species lacking sex chromosomes. Although we detected no effects on whole-organism traits under benign conditions, future work evaluating a broader range of fitness traits under stressful conditions may reveal unmeasured consequences.

## Supporting information

S1 Fig Sex specific results of *T. californicus* assayed for relative mitochondrial DNA content (a), 8-OH-dG DNA damage estimated by ELISA (b), routine metabolic rates estimated as oxygen consumed per picomole per hour then size corrected to protein content (c), and protein content (d). (TIFF)

S1 File Spreadsheet of data used in this manuscript. (XLSX)

S2 File R code used in analysis of data for this manuscript. (RMD)
