# Supplementary figures and images for: Molecular consequences of mitochondrial replacement may be masked from organismal traits in Tigriopus californicus
Source: PLoS One. 2025 Oct 24;20(10):e0335181. doi: 10.1371/journal.pone.0335181 (PMC12551816; doi:10.1371/journal.pone.0335181)

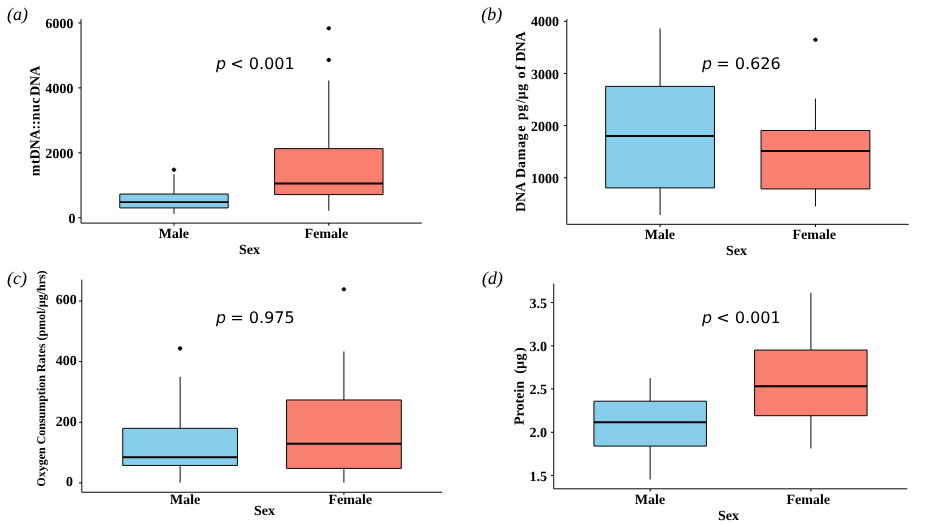

Supplement: S1 Fig — (TIFF) [file pone.0335181.s001.tiff]
